# Which Are the Cells of Origin in Merkel Cell Carcinoma?

**DOI:** 10.1155/2012/680410

**Published:** 2012-12-13

**Authors:** Thomas Tilling, Ingrid Moll

**Affiliations:** Department of Dermatology, University Medical Center Hamburg-Eppendorf, Martinistraße 52, 20246 Hamburg, Germany

## Abstract

Merkel cell carcinoma (MCC), a highly aggressive skin tumour with increasing incidence, is associated with the newly discovered Merkel cell polyomavirus (MCPyV). Studies on MCC and MCPyV as well as other risk factors have significantly increased our knowledge of MCC pathogenesis, but the cells of origin, which could be important targets in future therapies, are still unknown. Merkel cells (MCs), the neuroendocrine cells of the skin, were believed to be at the origin of MCC due to their phenotypic similarities. However, for several reasons, for example, heterogeneous differentiation of MCCs and postmitotic character of MCs, it is not very likely that MCC develops from differentiated MCs. Skin stem cells, probably from the epidermal lineage, are more likely to be cells of origin in MCC. Future studies will have to address these questions more directly in order to identify the physiological cells which are transformed to MCC cells.

## 1. Introduction

Merkel cell carcinoma (MCC) is a highly aggressive skin malignancy mainly affecting elderly and immunosuppressed people [1]. MCC is a rare tumour, but its incidence, especially in men, has risen during the last two decades in several countries [2]. For instance, an annual increase of 8% between 1986 and 2001 has been reported for the United States [3], and in the Netherlands, MCC incidence rates have doubled between 1993 and 2007 [4]. In Scandinavia, however, incidence of MCC did not rise between 1995 and 2005 [2]. MCC owes its name to characteristics it shares with Merkel cells (MCs), the neuroendocrine cells of the skin [5, 6]. Interest in MCC has significantly increased during the last years due to the discovery of Merkel cell polyomavirus (MCPyV) and its implication in MCC pathogenesis [7–9]. Studies on MCPyV and other molecular risk factors like mutations in the *ATOH1* gene [10] have greatly advanced our understanding of MCC pathogenesis. The monoclonal integration of viral DNA in a large proportion of MCCs suggests that viral infection precedes tumourigenesis [7, 11]. Furthermore, MCPyV T antigens are required for maintenance of virus-carrying MCC cell lines [12]. Binding of large T antigen to cell cycle regulators or of small T antigen to the translation regulator 4E-BP1 are potential mechanisms by which integrated MCPyV could transform cells [11, 13]. MCPyV may thus at least contribute to MCC formation, being the first molecular risk factor identified in MCC [9]. Additionally, mutations in several genes, including the tumour suppressor *ATOH1*, encoding a transcription factor involved in Merkel cell differentiation, and *PIK3CA*, which codes for phosphatidylinositol 4,5-bisphosphate 3-kinase, catalytic subunit, alpha isoform, have been found in subsets of MCC [10, 14]. Such mutations might either act in concert with MCPyV during tumourigenesis, or cause MCC formation in the absence of the virus. However, the question for the cell of origin in MCC remains unresolved. If it was known from which cell type MCC originates, one could develop more specific therapies which target these particular cells. In the present paper, we therefore reconsider potential cells of origin in MCC. We start with a brief discussion of cells of origin in cancer and subsequently elaborate on different cell types which have been hypothesized to be at the origin of MCC. Last but not least, we briefly discuss how MCPyV might affect the potential MCC precursors.

## 2. Cells of Origin in Cancer

Cells of origin in cancer have been defined as cells which “acquire the first genetic hit or hits that culminate in the initiation of cancer” [15]. Given the extended lifetime and self-renewal of physiological stem cells, these cells which assure homeostasis of rapidly self-renewing tissues—like skin—appear particularly prone to accumulate oncogenic mutations [15–17]. Therefore, it is not surprising that various studies have identified stem cells as cells of origin in cancer, for example, hematopoietic stem cells in chronic myeloid leukemia (CML) [18] or crypt stem cells in intestinal cancer [19]. In the skin, Youssef et al. [20] identified long-term resident progenitor cells of the interfollicular epidermis and the upper infundibulum as cells of origin in basal cell carcinoma (BCC), using clonal analysis in mice. These long-lived progenitor cells could, however, also be called “epidermal stem cells” according to Sellheyer [21]. It is noteworthy that this stem cell population is also the cell of origin of Merkel cells in mice [22–24]. Furthermore, squamous cell carcinoma (SCC) could be induced in hair follicle stem cells as well as in cells immediately exiting the bulge, but not in transit amplifying cells, which are more developmentally restricted [25, 26]. Generally, it should be noted that the terms “stem cells” and “progenitor cells” are often used interchangeably, as a sharp distinction is not always easy.

The second group of cells of origin in cancer are committed progenitor cells [15] which differ from stem cells by their much more restricted differentiation potential. Such cells play an important role in the acute phase of CML [27], but have also been identified as cells of origin in solid tumours. Examples include medulloblastoma arising from granule neuron progenitors [28, 29] and breast cancer developing from luminal epithelial progenitors [30]. 

Third, even differentiated cells could give rise to cancer, as any cell which is able to proliferate could become a cell of origin in cancer, provided it acquires mutations which restore the ability to self-renew and prevent differentiation to a postmitotic state [15]. For instance, a study in mice strongly suggests that malignant peripheral nerve sheath tumours arise from differentiated glia [31]. Moreover, differentiated endocrine cells in the pancreas appear to be a target for oncogenic transformation [32]. However, it should be noted that in both cases, less differentiated progenitor cells cannot be definitively excluded as cells of origin [15].

Before turning to the origin of MCC, it is important to keep in mind that the terms “cell-of-origin in cancer” and “cancer stem cell” are different [15, 33]. The “cell-of-origin” is a physiological cell which becomes tumourigenic due to genetic alterations. By contrast, “cancer stem cells” are a cell population within a tumour which constantly self-renews and is able to generate all types of cells present in this tumour, thus preserving the tumour. Briefly, the “cell-of-origin” *acquires* tumourigenic properties, whereas the “cancer stem cell” *sustains* tumourigenic properties. Regarding the cancer stem cell concept, the reader is referred to recently published excellent review articles [33–35]. 

## 3. Putative Cells of Origin for MCC

### 3.1. Merkel Cells

**Figure 1:**
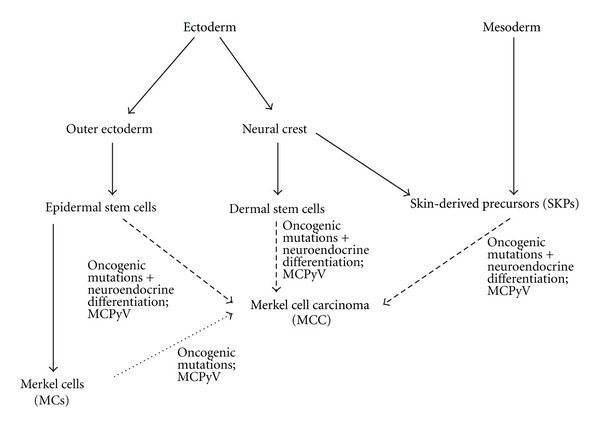
Scheme of potential cells of origin of Merkel cell carcinoma (MCC), shown from an ontogenetic perspective. All arrows with a *scattered*  line represent thus far hypothetic lineage relationships. However, whereas MCC derivation from Merkel cells is not very likely, there are hints implying epidermal stem cells in MCC genesis, and dermal stem cells as well as SKPs at least cannot be excluded as cells of origin for MCC. For each putative cell type of origin, an involvement of MCPyV in MCC genesis is highly probable, at least in a large fraction of MCCs.

Early histological and ultrastructural analyses of the so-called “trabecular carcinoma of the skin” [36, 37] revealed similarities to Merkel cells [5, 6, 36], leading to the currently used designation “Merkel cell carcinoma” [5, 6]. Moreover, the reported morphological observations led to the conclusion that MCC may most probably originate from Merkel cells (MCs) [5, 6, 36]. This traditional view of MCC origin (Figure 1) was further corroborated by the discovery that MCC and MCs share a similar immunophenotype [1, 38]. Shared features include presence of the Merkel cell marker cytokeratin 20 (CK20) in MCC [39, 40] as well as biosynthesis of synaptophysin [41–44], NCAM/CD56 [41, 45, 46], and numerous endocrine markers [38].

Although at first glance, these observations strongly support the hypothesis that MCC emerges from transformed MCs, there are quite a few data which question this view. First, MCs and MCC differ in some aspects of their immunophenotype. For instance, the neural cell adhesion molecule L1 (CD171), a relative of NCAM, is produced by MCC cells, but not by MCs [47]. Moreover, the arrangement of intermediate filaments, including CK20 and neurofilaments, differs between MCC and MCs: in MCCs, whirl-or plaque-like aggregates are observed, whereas in MCs, the intermediate filament cytoskeleton is loosely and diffusely arranged [48]. Last but not least, the tyrosine kinase receptor c-kit, which has been detected in the majority of MCCs [49], is mostly absent from human MCs [48].

Second, in a study on fetal and human skin, no proliferative Merkel cells could be detected, strongly suggesting that human Merkel cells are postmitotic [50]. In line with these findings, more recent lineage tracing analyses revealed that in mice, adult Merkel cell homeostasis is ensured by differentiation of epidermal progenitors, not through the proliferation of differentiated MCs [22]. Given that the possibility to restore proliferative potential is a prerequisite for a cell to become cell-of-origin in cancer [15], it is highly unlikely that postmitotic MCs would be the source of a malignancy.

Third, differences in tissue localization argue against MCs as cells-of-origin for MCC. Whereas the majority of MCs is located in the basal layer of the epidermis [48], MCCs are mostly found in the dermis and subcutis [51]. However, it should be noted that a minority of 3.2% to 9.1% of reported cases are partly or even fully localized in the epidermis [1]. The “localization argument” thus has to be handled with care.

Finally, the observed heterogeneity in MCC rather favors less differentiated cells as cells of origin [1]. In detail, MCCs associated with diverse differentiation patterns have been described: squamous [52], squamous and sarcomatous [53], melanocytic [54], eccrine [55], leiomyosarcomatous [56], rhabdomyoblastic [57], and fibrosarcomatous [58] differentiation. Although there is a certain immunophenotypical diversity in MCs themselves [41], the multitude of differentiation patterns in MCC rather points to stem or early progenitor cells as cells-of-origin. Such cells would possess the potential to differentiate along different lineages. It is, of course, conceivable that distinct stem or progenitor populations account for MCCs with distinct differentiation patterns. In the following sections, we will therefore discuss stem cell populations in skin which might serve as cell of origin in MCC.

### 3.2. Epidermal Stem Cells

As mentioned before, in mice, MCs arise from stem/progenitor cells of the epidermal lineage [22–24]. Therefore, epidermal stem cells or long-lived epidermal progenitor cells appear to be good candidates for a MCC cell-of origin (Figure 1). Along this line, Lemasson et al. have detected CK14-positive cells in MCC samples [59]. As CK14 is a marker of the basal epidermal layer which comprises the epidermal stem cells [60], and as MCs in mice have CK14-positive progenitors [22–24], the authors argued that a malignant transformation of epidermal stem cells could underlie MCC, and that the transformed cells could then serve as cancer stem cells for MCC [59]. In the same study, nestin immunoreactivity was detected in 20–30% of MCC tumoural cells. Nestin is regarded as a marker of multilineage progenitor cells [61]. However, contradictory to the findings of Lemasson et al., Abbas and Bhawan [62] reported the absence of nestin in all 11 MCC cases investigated. It cannot be excluded that these discrepancies were caused by different immunostaining protocols, as high variability of nestin immunoreactivity in skin due to procedural differences has been described [63]. Nevertheless, both studies showed the presence of CK19-positive cells in MCC [59, 62]. CK19 has been used as an epidermal stem cell marker [64], but is also present on mature Merkel cells [65]. Moreover, Abbas and Bhawan found no CK15-positive cells in MCC [62]. This might be an argument against hair follicle bulge stem cells as cells of origin in MCC, as CK15 is found in these cells [66]. Summarizing, there are several hints pointing to an epidermal stem/progenitor cell origin of MCC, but no experimental proof so far.

### 3.3. Dermal Stem Cells Derived from the Neural Crest

Based on their histomorphological pattern as well as results of immunohistochemical stainings, dermal neuroendocrine cells have been suggested as a potential source for MCC [51, 67]. This suggestion points to a second stem cell population present in skin, dermal stem cells (DSCs) derived from the neural crest lineage [68, 69] (Figure 1). Interestingly, the transcription factor Sox2, which is expressed by neural crest-derived stem cells from human skin [70], has been detected in MCC by immunohistochemistry [71]. Nine MCC samples were all Sox2-positive, with more than 50% of cells exhibiting nuclear staining. On the other hand, Sox2 is expressed by epidermal progenitors in the mouse tongue during their differentiation to sensory cells [72], demonstrating that it cannot be regarded as an exclusive neural crest-derived stem cell marker. Therefore, although MCC cells express several proteins abundant in neural cells, strong hints to a neural crest origin of MCC are currently missing.

### 3.4. Skin-Derived Precursors

In addition to the neural crest-derived stem cells mentioned before, a further stem cell population has been isolated from murine and human dermis and termed skin-derived precursors (SKPs) [73, 74]. These cells are able to differentiate into both neural and mesodermal cell types. In mice, facial SKPs are generated from the neural crest, whereas dorsal trunk SKPs derive from the somites [75] (Figure 1). Although there are a lot of similarities between SKPs and the DSCs mentioned before, both cell types differ in that SKPs lack the robust expression of the neurotrophin receptor p75 which is characteristic for DSCs [69]. Given their dermal localization and their broad differentiation potential, SKPs should be regarded as a further potential cell of origin for MCC. 

## 4. MCC Cells of Origin and the MCPyV

If the MCPyV plays an important role in MCC genesis, as suggested by several recent studies (reviewed in [8, 9]), it should target the putative cells of origin (Figure 1). It is noteworthy that JC polyomaviruses have been shown to preferentially infect stem cells or progenitor cells with a low degree of differentiation [76]. If MCPyV exhibits a similar preference, this would argue for a stem rather than a Merkel cell at the origin of MCC. Along the same line, unpublished findings from our laboratory suggest that human Merkel cells are normally devoid of MCPyV large T antigen (LTAg). Among 733 MCs detected by CK20 immunostaining in human skin areas prone to MCC development, none exhibited LTAg immunopositivity in the adjacent section. Although this does not prove the absence of MCPyV from differentiated MCs, it makes them less likely as a target for MCPyV infection.

## 5. Summary and Conclusions

Despite intense research on MCC during the last years, the cells of origin of this cutaneous malignancy remain elusive. Merkel cells (MCs), originally the favourite candidate for such a role, appear less likely to give rise to MCC now, mainly because of MCC heterogeneity and the postmitotic character of MCs. Stem cells located in the skin, most probably of the epidermal lineage, appear to be more probable cells of origin for MCC. However, as experimental evidence for a stem cell origin is missing, too, more direct approaches to tackle the “origin-of-MCC-question” are needed. These could include genetic lineage tracing or reprogramming of MCC cells.
